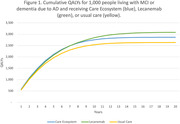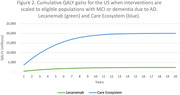# The Scale of Benefit and Cost Effectiveness of the Care Ecosystem for Alzheimer’s Disease relative to Lecanemab

**DOI:** 10.1002/alz.089579

**Published:** 2025-01-09

**Authors:** Kelly J Atkins, James G Kahn, Katherine L Possin

**Affiliations:** ^1^ University of California San Francisco, San Francisco, CA USA

## Abstract

**Background:**

Only a small proportion of people living with Alzheimer’s Disease (AD) are eligible for disease modifying therapies such as Lecanemab. Dementia care management programs, such as Care Ecosystem, provide care navigation and specialist resources to people with dementia and their caregivers, with broad eligibility criteria. We first estimated the lifetime health and economic outcomes of adults with MCI or mild dementia due to AD who receive Care Ecosystem, compared to Lecanemab. We then estimated the benefits of each intervention when scaled to the eligible US population.

**Method:**

We employed a decision analytic model to a simulation sample of 1,000 people (52% female) aged 71 years at the commencement of one of three intervention states: 1) usual care, 2) Lecanemab or 3) Care Ecosystem. Next, we applied our model outcomes to all people with MCI or dementia due to AD who were eligible for each intervention. Primary outcomes were quality‐adjusted life years (QALYs); remaining life years (LYs); and costs in 2024 US dollars.

**Result:**

Per‐person LYs increased by 0.39 per person with Lecanemab compared to Care Ecosystem and usual care. Lifetime QALYs for Lecanemab were 0.45 higher than usual care, due to disease slowing. Lifetime QALYs for the Care Ecosystem were 0.23 higher than usual care, due to improved quality of life (Figure 1). The per‐person cost of Lecanemab was $105,581 higher than usual care, driven primarily by drug costs. The per‐person cost of the Care Ecosystem was $11,304 lower than usual care, reflecting savings to Medicare associated with dementia care management. In the US in 2024, we estimated ∼765,000 people were eligible for Lecanemab and 6.1 million for Care Ecosystem. The population benefit of Lecanemab was 760,000 QALYs and cost $80 billion compared to usual care. The population benefit of Care Ecosystem was 6,160,000 QALYs with savings of $69 billion (Figure 2).

**Conclusion:**

Lecanemab offers a larger QALY gain per‐person than provided by Care Ecosystem. When scaled to eligible populations, the QALY gains of Care Ecosystem far exceed Lecanemab, with a net cost reduction. Universal access to dementia care management should be a priority of health care policy.